# Infectious bronchitis virus accessory protein 3a induces renal inflammatory injury by activating the NLRP3 inflammasome via ER calcium mobilization and mitochondrial ROS production

**DOI:** 10.1128/jvi.02125-25

**Published:** 2026-04-14

**Authors:** Min Huang, Chengyin Liukang, Huiming Yang, Lu Sun, Lihua Tang, Pinyi Lv, Feixuan Hou, Ye Zhao, Guozhong Zhang, Jing Zhao

**Affiliations:** 1 State Key Laboratory of Veterinary Public Health and Safety, College of Veterinary Medicine, China Agricultural University, Beijing, People’s Republic of China; 2 Key Laboratory of Animal Epidemiology of the Ministry of Agriculture, College of Veterinary Medicine, China Agricultural University, Beijing, People’s Republic of China; University of Kentucky College of Medicine, Lexington, Kentucky, USA

**Keywords:** infectious bronchitis virus, accessory protein 3a, NLRP3 inflammasome, ER calcium mobilization, mitochondrial ROS

## Abstract

Infectious bronchitis virus (IBV) causes severe renal injury in chickens. Previous work showed that IBV promotes renal pathology by activating the NLRP3 (NOD-like receptor family pyrin domain-containing 3)–Caspase-1IL-1β (interleukin-1 beta) axis in collecting duct epithelial cells. Here, we identify viral determinants and upstream signaling pathways that drive this response. We find that the accessory protein 3a interacts with NLRP3, promotes its oligomerization and enhances inflammasome assembly, thereby acting as a key effector. We generated a 3a-knockout virus (rSD-Δ3a) and a start codon substitution mutant (rSD-3a-GTG) and rescued these recombinant viruses. Compared with the parental rSD strain, these mutants reduced Caspase-1 activity and IL-1β maturation and release in chicken embryonic kidney (CEK) cells. *In vivo*, IBV mutants lacking 3a expression significantly attenuated NLRP3 inflammasome activation and proinflammatory cytokine production in renal tissue, mitigated renal pathology, and improved survival. Mechanistically, IBV infection promotes K^+^ efflux and Ca²^+^ mobilization, which together facilitate NLRP3 inflammasome activation. Protein 3a colocalizes with NLRP3 at the endoplasmic reticulum (ER), enhances ER Ca²^+^ release, and drives mitochondrial Ca²^+^ overload and accumulation of mitochondrial reactive oxygen species (mtROS), both required for NLRP3 activation. Collectively, these findings establish 3a as a viral driver of NLRP3-mediated pathology, advance understanding of IBV renal pathogenesis, and support the rational design of attenuated IBV vaccine strains.

**IMPORTANCE:**

Infectious bronchitis virus (IBV)-induced renal injury is associated with activation of the NLRP3 inflammasome in collecting duct epithelial cells, yet the mechanism by which IBV promotes inflammasome activation remains unclear. Here, we show that the accessory protein 3a interacts with NLRP3 and promotes its assembly, playing a pivotal role in activating the NLRP3 inflammasome and the subsequent renal injury. Specifically, 3a induces ER Ca²^+^ release and promotes accumulation of mitochondrial reactive oxygen species (mtROS), thereby enhancing NLRP3 inflammasome activation. These findings indicate that IBV 3a is a critical mediator of NLRP3-dependent inflammatory activation, advance our understanding of IBV renal pathogenesis, and provide a rationale and potential targets for the design of gene-deletion attenuated IBV vaccines.

## INTRODUCTION

Avian infectious bronchitis virus (IBV), a member of the genus Gammacoronavirus, causes a highly contagious respiratory disease in chickens and commonly leads to reduced egg production and quality, causing substantial economic losses (1–3). Currently, nephropathogenic IBV predominates in circulation and causes severe nephritis in chicks, urate deposition in the ureters, and increased mortality, substantially compromising flock health and production (4, 5). Although extensive descriptions of nephropathogenic IBV-associated renal lesions exist, the cellular and molecular mechanisms driving IBV-induced kidney injury remain unclear (6, 7).

The NOD-like receptor family pyrin domain-containing 3 (NLRP3) inflammasome is a key cytosolic signaling platform of the innate immune system that senses pathogen-associated molecular patterns and damage-associated molecular patterns (8–10). Its activation generally follows a two-signal model: priming (signal 1), in which upstream cues, such as toll-like or cytokine receptors, activate NF-κB to induce NLRP3 and pro-IL-1β (interleukin-1 beta) expression; and activation (signal 2), in which detection of exogenous pathogens or endogenous danger signals triggers NLRP3 conformational change and oligomerization, promoting inflammasome assembly and Caspase-1 activation and thereby driving maturation and release of IL-1β (interleukin-1 beta) and IL-18 (interleukin-18) (11–13). Canonical activating signals include K^+^ efflux, Ca²^+^ mobilization, lysosomal damage, and mitochondrial dysfunction accompanied by accumulation of mitochondrial reactive oxygen species (mtROS) (14–17). Physiologically, moderate NLRP3 activation supports pathogen clearance and tissue homeostasis. By contrast, persistent or excessive activation results in excessive IL-1β and IL-18 production and upregulation of downstream proinflammatory mediators, such as IL-6 (Interleukin-6), culminating in systemic hyperinflammation and, at the extreme, a cytokine storm—an association reported across multiple acute inflammatory diseases and viral infections (18–20). Thus, the NLRP3 inflammasome functions as a “double-edged sword” at the interface of antiviral immunity and inflammatory pathology (21).

Previous studies indicate that viruses are not merely passive targets of NLRP3 inflammasome recognition but also actively modulate NLRP3 activation through diverse mechanisms (22–24). Viruses can provide the activation signal (signal 2) by perturbing ionic homeostasis—including K^+^ efflux and Ca²^+^ mobilization—inducing lysosomal damage, and disrupting mitochondrial function and redox homeostasis; in some contexts, they can also modulate priming (signal 1) (25, 26). A prominent mechanism involves viral proteins that directly or indirectly interact with inflammasome components, promoting NLRP3 conformational change and oligomerization and thereby enhancing inflammasome assembly and downstream effector activation (27). For example, viral structural and accessory proteins can alter K^+^ efflux and Ca²^+^ mobilization, induce mitochondrial damage, or bind NLRP3 and its cofactors to modulate the IL-1β response and disease pathogenesis (28–31). The genome of infectious bronchitis virus encodes the structural proteins S, E, M, and N; the accessory proteins 3a, 3b, 5a, and 5b; and replication-associated non-structural proteins encoded by ORF1a/1b (32). Our previous work demonstrated that IBV infection activates the NLRP3–Caspase-1–IL-1β axis in chicken renal collecting duct epithelial cells, promoting IL-1β maturation and release and being associated with inflammatory kidney injury in chickens (33). Nevertheless, the specific viral effectors and upstream signaling events that mediate NLRP3 activation during IBV infection remain to be elucidated.

## RESULTS

### IBV accessory protein 3a induces punctate aggregation of NLRP3

**Fig 1:**
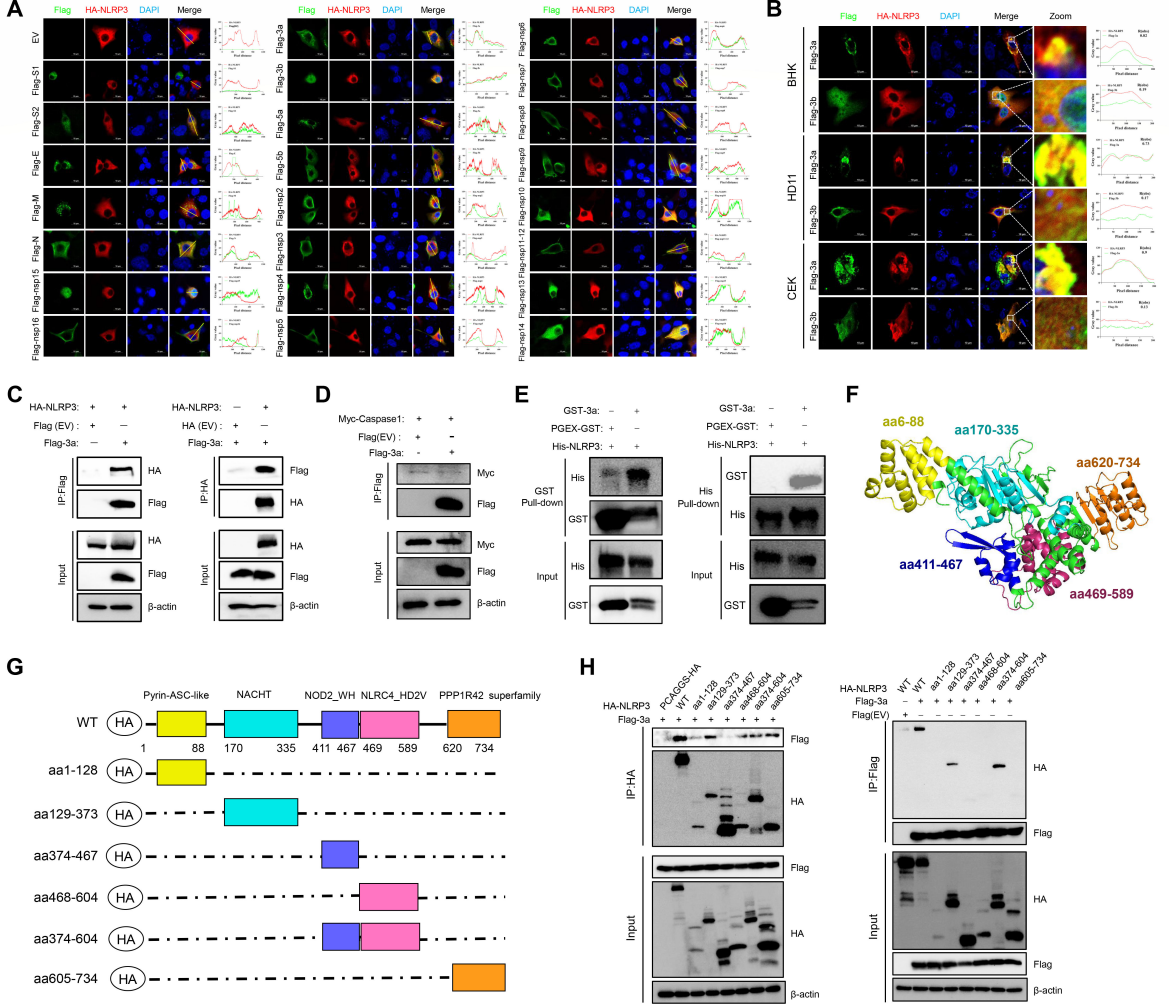
Interaction between IBV 3a protein and NLRP3 and identification of critical binding domains. (**A**) Screening of IBV proteins for induction of NLRP3 aggregation. BHK cells were co-transfected with HA-tagged NLRP3 and individual Flag-tagged IBV proteins. At 24 h post-transfection, subcellular localization of HA-NLRP3 (red) and Flag-IBV proteins (green) was assessed by confocal microscopy. Scale bar = 10 µm. (**B**) 3a-induced NLRP3 punctate aggregation in BHK, HD11, and CEK cells. Cells were co-transfected with HA-NLRP3 and either Flag-3a or Flag-3b (negative control) and examined 24 h later by confocal microscopy. HA-NLRP3 and Flag-tagged proteins are shown in red and green, respectively. Representative punctate aggregates are highlighted with white dashed boxes. Scale bar = 10 µm. (**C and D**) Co-IP analysis of interactions between IBV 3a and NLRP3 or Caspase-1. (**C**) HEK293T cells were co-transfected with Flag-3a and HA-NLRP3. Cell lysates were immunoprecipitated with anti-Flag or anti-HA antibodies, followed by western blot detection of co-immunoprecipitated proteins. (**D**) HEK293T cells were co-transfected with Flag-3a and Myc-Caspase-1 and processed as in panel C. Input represents total cell lysates before immunoprecipitation. (**E**) GST pull-down assay assessing direct interaction between 3a and NLRP3. Purified GST-3a or GST was incubated with recombinant His-NLRP3, and bound proteins were pulled down using GST agarose beads. In a reciprocal assay, His-NLRP3 was incubated with GST-3a and captured using His agarose beads. Bound proteins were analyzed by immunoblotting with anti-GST and anti-His antibodies. (**F**) Schematic of the AlphaFold-predicted structure of chicken NLRP3 (chNLRP3). (**G**) Domain organization of full-length chNLRP3 and its truncation mutants. (**H**) Mapping of 3a interactions with chNLRP3 truncation mutants by co-immunoprecipitation. HEK293T cells were co-transfected with the indicated plasmids. Cell lysates were immunoprecipitated with anti-Flag or anti-HA antibodies and analyzed by western blot. Input represents total lysates before immunoprecipitation.

Building on our previous finding that IBV activates the NLRP3 inflammasome in chicken renal collecting duct epithelial cells (33), we sought to further investigate the underlying mechanism of inflammasome activation. To elucidate this mechanism, we first systematically screened all IBV structural, non-structural, and accessory proteins for their ability to induce NLRP3 discrete cytosolic foci. The formation of NLRP3 discrete cytosolic foci signifies the activation and assembly of the inflammasome, which is a key step in its functional maturation. Confocal microscopy revealed that accessory protein 3a uniquely induced perinuclear NLRP3 transition from diffuse cytosol to punctate structures when co-expressed with NLRP3 (Fig. 1A). In addition to BHK cells, similar NLRP3 transition from diffuse cytosol to punctate structures was also observed in HD11 cells (a chicken macrophage-like cell line) and chicken embryonic kidney (CEK) cells upon 3a expression (Fig. 1B). Co-immunoprecipitation (Co-IP) assays showed that 3a specifically interacts with NLRP3 but not with Caspase-1, while pull-down assays confirmed a direct interaction between 3a and NLRP3 (Fig. 1C through E).

Guided by the AlphaFold-predicted structure of chicken NLRP3 (chNLRP3) (Fig. 1F), we generated a series of truncation mutants for domain mapping (Fig. 1G). Co-IP and confocal imaging revealed that 3a interacts with the NACHT, NLRC4-HD2V-like, and PPP1R42-like domains, promoting NLRP3 punctate aggregation (Fig. 1H; Fig. S1). These findings establish that IBV 3a promotes NLRP3 oligomerization and punctate aggregate formation by targeting specific domains of chNLRP3, highlighting its central role in inflammasome assembly.

### IBV 3a triggers NLRP3-dependent inflammation-induced renal injury

**Fig 2:**
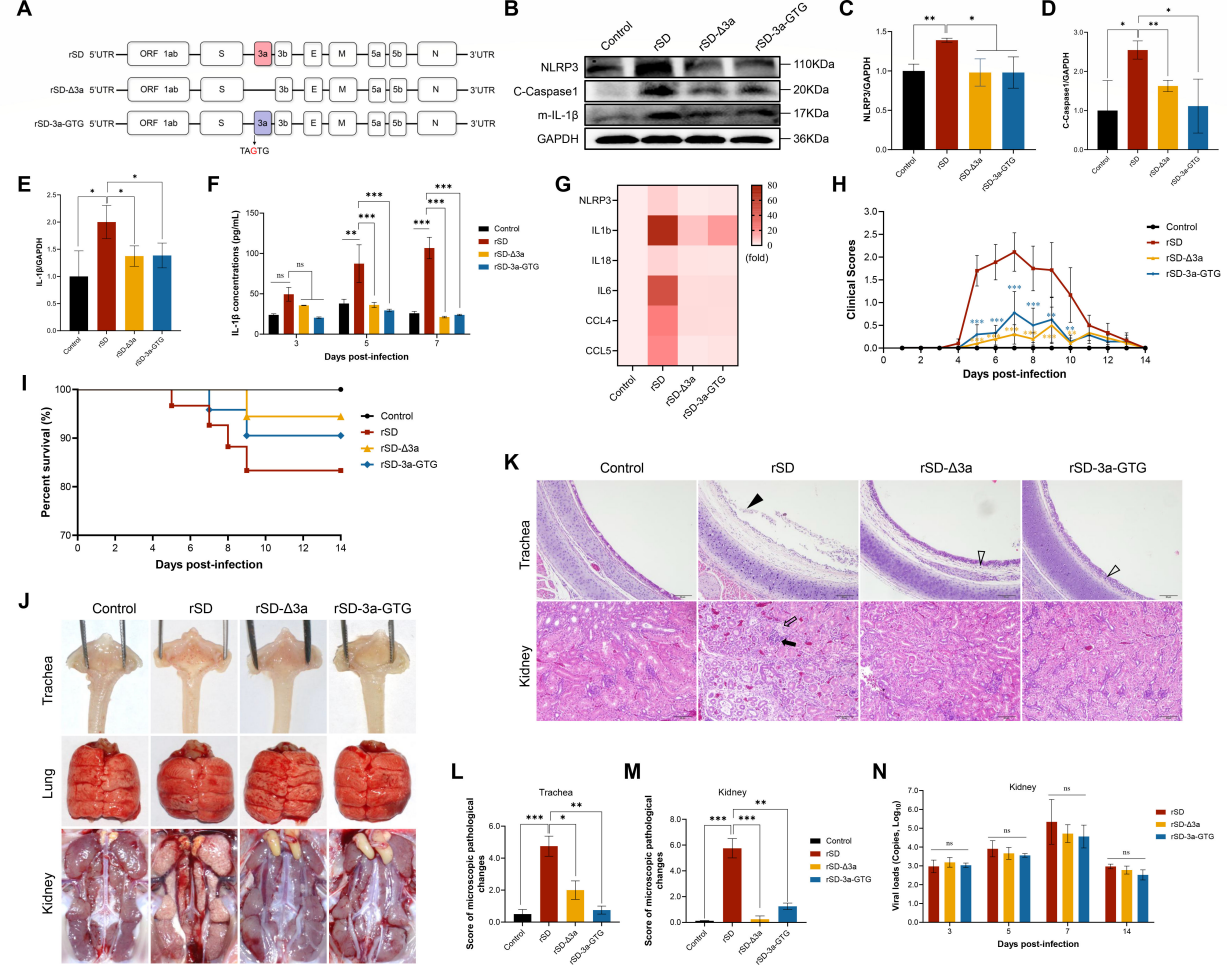
Loss of 3a expression attenuates inflammasome activation and viral pathogenicity. (**A**) Schematic of the design and genomic mutation sites of rSD-Δ3a and rSD-3a-GTG recombinant viruses. (**B–E**) Assessment of NLRP3 inflammasome activation in the kidneys of chickens infected with IBV recombinant strains. Renal tissues at 5 dpi were collected for western blot analysis of NLRP3, cleaved Caspase-1, and mature IL-1β. (**B**) Representative immunoblots. (C–E) Quantification of the indicated proteins. β-actin served as a loading control. (**F**) ELISA quantification of serum IL-1β levels at 5 dpi. (**G**) qRT-PCR of renal inflammatory genes after IBV infection. 3a deletion or mutation reduced *NLRP3, IL1B, IL18, IL6, CCL4*, and *CCL5* expression. Expression was normalized to β-actin. (**H**) Daily clinical sign scores for each experimental group, according to the scoring criteria provided in Fig. 3H legend. (**I**) Survival curves for each group over a 14-day observation period. (**J**) Gross pathology of the trachea, lungs, and kidneys on day 5 post-infection across experimental groups. (**K**) H&E-stained sections of tracheal and renal tissues from each group. Black arrowheads denote detachment, degeneration, and necrosis of tracheal ciliated epithelial cells; open black arrowheads represent mild inflammatory cell infiltration; solid black arrows indicate interstitial inflammatory cell infiltration in renal tissue; open black arrows highlight vacuolar degeneration and necrosis of renal tubular epithelial cells. Scale bar = 50 µm. (**L and M**) Histopathological injury scores for tracheal and renal tissues, with scoring criteria described in Fig. 3L legend. (**N**) Renal IBV viral load at 5 dpi was determined by qRT-PCR targeting viral RNA from each group. All data are presented as mean ± SD, *n* = 3. ns, not significant; *, *P* < 0.05; **, *P* < 0.01; ***, *P* < 0.001.

To elucidate the role of the IBV accessory protein 3a in NLRP3 inflammasome activation and renal injury, we generated a recombinant 3a gene knockout virus (rSD-Δ3a) and a start codon substitution mutant (rSD-3a-GTG, in which the original ATG was replaced by GTG), and subsequently rescued these recombinant viruses (Fig. 2A). Both rSD-Δ3a and rSD-3a-GTG induced typical chicken embryo lesions and exhibited replication kinetics similar to the parental rSD strain in CEK cells and embryonated chicken eggs (Fig. S2A and B), indicating that 3a is dispensable for viral replication.

The regulatory role of the IBV accessory protein 3a in NLRP3 inflammasome activation was first assessed *in vitro* using CEK cells. At 24 h post-infection (hpi), both rSD-Δ3a and rSD-3a-GTG significantly reduced NLRP3, pro-IL-1β expression, along with decreased release of mature IL-1β (Fig. S2C). Caspase-1 activity and IL-1β release were also markedly reduced (Fig. S2D and E).

To validate these findings *in vivo*, 1-day-old specific-pathogen-free (SPF) chickens were challenged with recombinant viruses to assess the role of 3a during infection. At 5 days post-infection (5 dpi), compared to rSD, chickens infected with 3a-deficient IBV strains exhibited markedly reduced renal expression of NLRP3, cleaved Caspase-1, and mature IL-1β, along with lower serum IL-1β levels and downregulated transcription of inflammasome-related cytokines and chemokines (Fig. 2B through G). During the 14-day observation period, chickens infected with rSD-Δ3a or rSD-3a-GTG showed milder clinical signs, while those infected with rSD developed typical IBV symptoms, including sneezing, head shaking, open-mouth breathing, and lethargy, beginning at day 5 and peaking at day 7 (Fig. 2H). Mortality was reduced in the rSD-Δ3a (5.56%) and rSD-3a-GTG (9.49%) groups compared to the rSD group (16.67%) (Fig. 2I). Gross pathological examination (Fig. 2J) revealed severe renal lesions, characterized by swelling and urate deposition, in the rSD group. Additionally, punctate hemorrhages and catarrhal exudates were observed in the throat and trachea. In contrast, chickens infected with 3a-deficient strains exhibited milder gross lesions in both the trachea and kidneys. Notably, no apparent macroscopic changes were observed in the lungs of any group. Histopathological analysis (Fig. 2K) revealed prominent renal lesions in the rSD group, characterized by tubular necrosis and inflammatory cell infiltration. Tracheal lesions, such as ciliary loss and epithelial necrosis, were also observed. While the 3a-deficient viruses caused significantly milder disease, the tracheal histopathological results revealed some mild residual lesions, including inflammatory cell infiltration and lamina propria edema. These lesions were less severe compared to the rSD group but still present in the 3a-deficient groups. In contrast, chickens infected with 3a-deficient strains exhibited significantly less severe lesions, particularly in the kidneys. Consistent with these observations, lesion scoring (Fig. 2L and M) indicated less severe renal and tracheal lesions in chickens infected with 3a-deficient strains. Importantly, the attenuation of tissue injury occurred without significant changes in renal viral RNA levels across groups (Fig. 2N), indicating that 3a contributes to tissue damage independently of viral replication. These findings collectively demonstrate that IBV 3a activates the NLRP3 inflammasome to drive renal inflammatory injury, underscoring its pivotal role in IBV-induced inflammation.

### Potassium efflux and calcium mobilization mediate IBV-induced NLRP3 inflammasome activation

**Fig 3:**
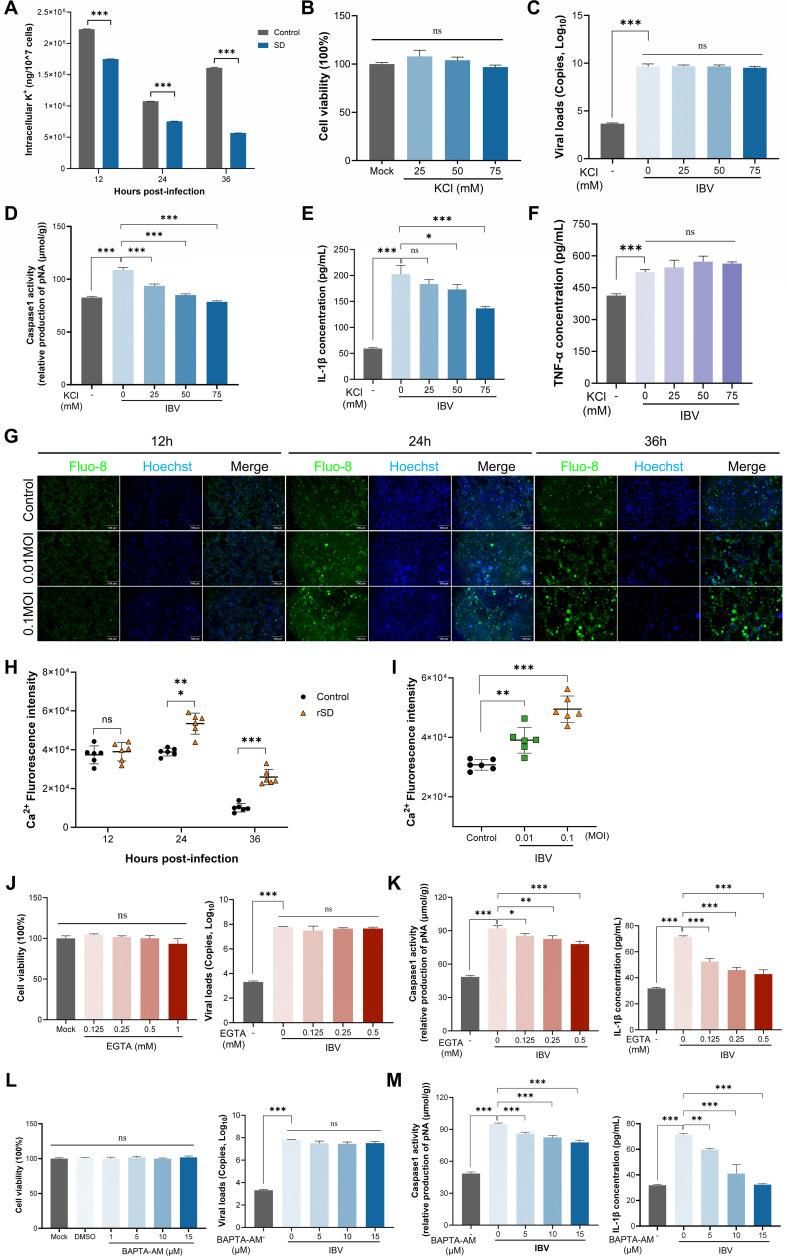
Effects of potassium efflux and calcium mobilization on NLRP3 inflammasome activation during IBV infection. (**A**) CEK cells were infected with IBV (MOI = 0.1), and intracellular potassium levels were quantified at 12, 24, and 36 hpi using ICP-MS. (**B–F**) CEK cells were infected with IBV in the presence of 25, 50, or 75 mM KCl and incubated for 24 h. Cell viability (**B**), viral RNA levels (**C**), Caspase-1 activity (**D**), and release of IL-1β (**E**) and TNF-α (**F**) were assessed. (**G–I**) IBV infection led to increased intracellular Ca²^+^ levels in CEK cells. (**G**) Cells were stained with Fluo-8 and observed by fluorescence microscopy, showing progressively increased Ca²^+^ fluorescence at 12, 24, and 36 hpi. (**H, I**) Quantification using a multi-mode microplate reader confirmed a time-dependent (**H**) and MOI-dependent (**I**) increase in intracellular Ca²^+^ levels. (**J–M**) To assess the role of calcium signaling in IBV-induced NLRP3 inflammasome activation, CEK cells were pretreated with varying concentrations of EGTA or BAPTA-AM for 2 h prior to infection (MOI = 0.01), followed by continued treatment for 24 h. Cell viability and viral RNA levels (**J and L**); Caspase-1 activity and IL-1β release (**K and M**) were measured using CCK-8, qRT-PCR, a Caspase-1 activity assay, and ELISA, respectively. All data are presented as mean ± SD, *n* = 3. ns, not significant; *, *P* < 0.05; **, *P* < 0.01; ***, *P* < 0.001.

It is well established that K^+^ efflux and Ca²^+^ mobilization are essential for NLRP3 inflammasome activation (34, 35). These ionic changes are sensed by the cell as stress signals, triggering the activation of the inflammasome. To investigate the role of potassium signaling in NLRP3 inflammasome activation during IBV infection, we measured intracellular potassium concentrations in CEK cells at 12, 24, and 36 hpi using inductively coupled plasma mass spectrometry (ICP-MS). IBV infection led to a significant decrease in intracellular potassium levels (Fig. 3A), suggesting that potassium efflux may act as an upstream signal for NLRP3 inflammasome activation. To assess the role of potassium efflux in IBV-induced NLRP3 activation, infected CEK cells were treated with 25, 50, or 75 mM KCl. This treatment significantly reduced Caspase-1 activity and IL-1β release without affecting viral replication, cell viability, or TNF-α levels (Fig. 3B through F). Potassium efflux appears to function as a key upstream signal for NLRP3 inflammasome activation during IBV infection.

We next explored the contribution of calcium signaling to IBV-induced NLRP3 inflammasome activation. Intracellular Ca²^+^ levels in infected CEK cells were measured using the calcium-sensitive dye Fluo-8, revealing a significant, time- and multiplicity of infection (MOI)-dependent increase (Fig. 3G through I). To investigate the roles of extracellular and intracellular calcium in the activation of the NLRP3 inflammasome, infected cells were treated with EGTA, an extracellular Ca²^+^ chelator, or BAPTA-AM, a cell-permeable intracellular Ca²^+^ chelator. Both inhibitors dose-dependently reduced Caspase-1 activation and IL-1β release, with BAPTA-AM showing a stronger effect, while neither treatment affected cell viability or viral replication (Fig. 3J through M). The findings suggest that both potassium efflux and calcium mobilization contribute to NLRP3 inflammasome activation during IBV infection.

### IBV 3a protein activates the NLRP3 inflammasome via increased ER Ca^2+^ efflux

**Fig 4:**
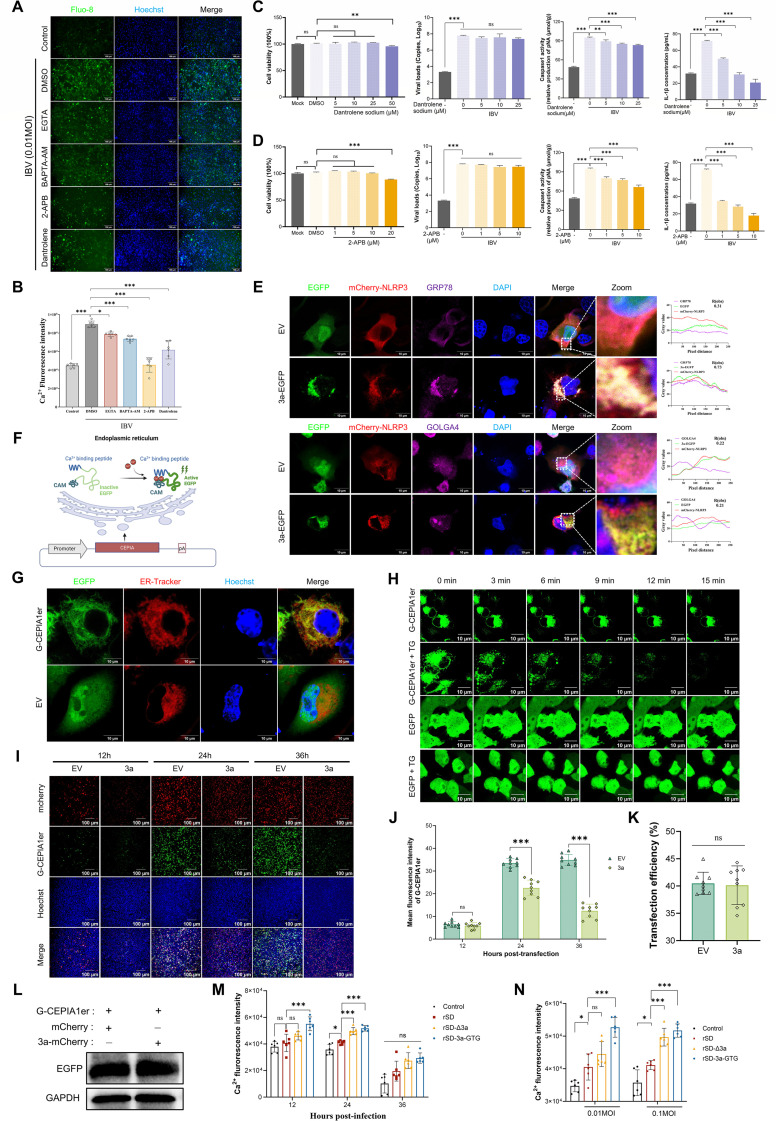
IBV 3a protein disrupts ER calcium homeostasis and facilitates NLRP3 inflammasome activation. (A–D) IBV-induced NLRP3 inflammasome activation requires ER-mediated Ca²^+^ signaling. (**A and B**) CEK cells were pretreated for 2 h with a panel of Ca²^+^ signaling inhibitors, then infected with IBV (MOI = 0.01) and incubated with the inhibitors for another 24 h. Cytosolic Ca²^+^ was detected by Fluo-8 AM fluorescence microscopy (**A**) and quantified with a multi-mode plate reader (**B**). (**C and D**) Caspase-1 activity (**C**) and IL-1β release (**D**) were measured using a fluorometric Caspase-1 activity assay and ELISA, respectively. (**E**) BHK cells were co-transfected with 3a-EGFP and mCherry-NLRP3 plasmids. After 24 h, immunofluorescence staining and confocal microscopy were used to assess 3a and NLRP3 co-localization. GRP78 and GOLGA4 served as ER and Golgi markers, respectively. (**F**) Schematic of ER-targeted Ca²^+^ probe G-CEPIA1er design and plasmid construction. Image created with BioRender. (**G**) BHK cells were transfected with the G-CEPIA1er plasmid and incubated for 24 h, then stained with ER-Tracker to label the endoplasmic reticulum. Co-localization was assessed by confocal microscopy. (**H**) Live-cell imaging of ER Ca²^+^ dynamics using the G-CEPIA1er probe. TG treatment induced a marked decrease in G-CEPIA1er fluorescence intensity, indicative of ER Ca²^+^ store depletion. In contrast, no significant fluorescence changes were observed in EGFP-expressing or untreated control cells. (**I–L**) Fluorescence imaging (**I**) and quantification of G-CEPIA1er fluorescence intensity (**J**) were performed in BHK cells co-expressing G-CEPIA1er and IBV 3a at 12, 24, and 36 h post-transfection. Transfection efficiency (**K**) and G-CEPIA1er expression levels (**L**) were assessed in control cells. (**M and N**) Cytosolic Ca²^+^ levels in CEK cells infected with recombinant IBV were assessed using the Fluo-8 AM probe. TG-induced ER Ca²^+^ release served as a functional indicator of ER Ca²^+^ storage. Panel **M** depicts results across varying infection durations, while panel **N** illustrates responses under different MOIs, both presented as baseline-corrected TG-induced fluorescence increases. All data are presented as mean ± SD. ns, not significant; *, *P* < 0.05; **, *P* < 0.01; ***, *P* < 0.001.

Building on our finding that Ca²^+^ signaling is essential for IBV-induced NLRP3 activation, we investigated the source of Ca²^+^ mobilization. To distinguish extracellular influx from intracellular release, infected CEK cells were treated with EGTA, BAPTA-AM, 2-APB (IP₃ receptor antagonist), or dantrolene (ryanodine receptor inhibitor). Fluo-8 AM imaging revealed reduced cytosolic Ca²^+^ levels with all treatments, with 2-APB and dantrolene showing stronger effects than EGTA (Fig. 4A and B). Inhibition of ER Ca²^+^ release significantly reduced Caspase-1 activity and IL-1β release (Fig. 4C and D), indicating that the ER is a major source of intracellular Ca²^+^ and a key contributor to NLRP3 activation during IBV infection.

Considering the ER’s central role in intracellular Ca²^+^ storage and inflammatory signaling (36, 37), we examined the subcellular localization of IBV 3a and NLRP3 using confocal microscopy. Confocal imaging showed extensive colocalization of IBV 3a and NLRP3 in the ER in BHK cells (Fig. 4E), as well as in avian HD11 and CEK cells (Fig. S3A). This consistent localization across species and cell types supports a potential role for 3a in modulating ER-related Ca²^+^ signaling. To test this, we constructed a plasmid encoding the ER-targeted Ca²^+^ probe G-CEPIA1er (Fig. 4F). Fluorescence imaging confirmed ER localization of G-CEPIA1er (Fig. 4G). Upon treatment with thapsigargin (TG), an inhibitor of sarco/endoplasmic reticulum Ca²^+^-ATPase (SERCA), G-CEPIA1er fluorescence gradually declined due to ER Ca²^+^ depletion, while EGFP remained stable (Fig. 4H; Movies S1 to S4), validating its reliability for monitoring ER Ca²^+^ dynamics.

We assessed the effect of 3a on ER Ca²^+^ release by co-expressing 3a and G-CEPIA1er in cells and performing confocal imaging at 12, 24, and 36 h to measure fluorescence intensity. 3a expression significantly reduced G-CEPIA1er fluorescence compared to the EV control, in a time- and dose-dependent manner (Fig. 4I and J; Fig. S3B and C). Similar transfection efficiency and G-CEPIA1er expression in 3a and control cells (Fig. 4K and L) suggest that fluorescence loss was due to 3a-induced ER Ca²^+^ mobilization. To further examine ER Ca²^+^ dynamics, cells were treated with TG to induce ER Ca²^+^ depletion. Cytosolic Ca²^+^ transients were monitored using Fluo-8 and compared among cells infected with rSD, rSD-Δ3a, or rSD-3a-GTG. Calcium responses were mainly affected by treatment duration, with minimal influence from viral strain (Fig. S3D through F). Based on these observations, a 1-min TG incubation was used as the standard stimulation protocol. TG increases cytosolic Ca²^+^ by blocking SERCA, triggering calcium release from the ER. The resulting cytosolic Ca²^+^ elevation indirectly reflects the amount of calcium stored in the ER prior to stimulation. TG-treated rSD-infected cells displayed markedly lower Fluo-8 fluorescence than rSD-Δ3a and rSD-3a-GTG cells, with the difference amplified by prolonged infection or higher MOI (Fig. 4M and N). These findings demonstrate that ER Ca²^+^ levels are markedly reduced in rSD-infected cells relative to rSD-Δ3a and rSD-3a-GTG controls, consistent with a role for the 3a protein in driving ER Ca²^+^ efflux. Together with G-CEPIA1er imaging and TG-induced Ca²^+^ release assays, these findings support that IBV 3a promotes ER Ca²^+^ efflux and contributes to NLRP3 inflammasome activation.

### IBV 3a-mediated ER Ca²^+^ release leads to mitochondrial calcium overload

**Fig 5:**
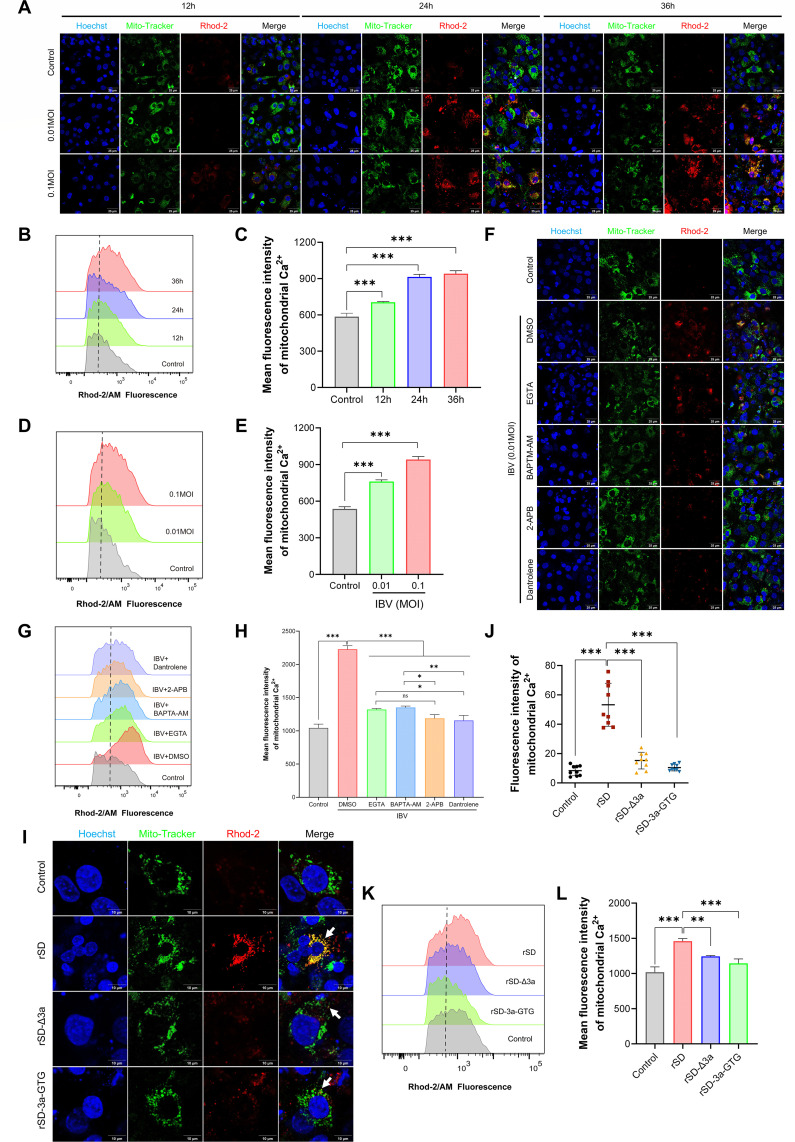
Mitochondrial Ca²^+^ accumulation in IBV-infected CEK cells. (**A–E**) Mitochondrial Ca²^+^ levels in CEK cells after IBV infection. (**A**) Cells were stained with Rhod-2 AM and imaged by confocal microscopy. (**B–E**) Mitochondrial Ca²^+^ levels were measured by flow cytometry in a time-dependent manner (**B**) and in response to increasing MOIs (**D**) with corresponding quantitative analyses shown in panels **C** and **E,** respectively. (**F–H**) Effects of calcium signaling inhibitors on IBV-induced mitochondrial Ca²^+^ accumulation. IBV-infected CEK cells were treated with EGTA, BAPTA-AM, 2-APB, or dantrolene. Mitochondrial Ca²^+^ was visualized by Rhod-2 AM staining and confocal microscopy (**F**), quantified by flow cytometry (**G**), and statistically summarized (**H**). (**I–L**) Effects of recombinant IBV strains on mitochondrial Ca²^+^ accumulation. CEK cells were infected with rSD, rSD-Δ3a, or rSD-3a-GTG. Cells were then stained with Rhod-2 AM and analyzed by confocal microscopy (**I and J**) and flow cytometry (**K and L**) to assess strain-dependent differences. *, *P* < 0.05; **, *P* < 0.01; ***, *P* < 0.001.

As the ER serves as the primary source of Ca²^+^ release during IBV infection and mitochondria act as key buffers to maintain intracellular calcium homeostasis (38, 39), it is possible that mitochondrial calcium handling is disrupted. To test this, we assessed whether IBV infection results in mitochondrial Ca²^+^ accumulation. Mitochondrial Ca²^+^ levels in IBV-infected CEK cells were measured using Rhod-2 AM, a mitochondria-targeted Ca²^+^-sensitive probe. Confocal microscopy and flow cytometry showed significant time- and dose-dependent increases in fluorescence, indicating mitochondrial Ca²^+^ accumulation and potential overload (Fig. 5A through E).

To identify the source of IBV-induced mitochondrial Ca²^+^ accumulation, infected CEK cells were pretreated with EGTA, BAPTA-AM, or ER Ca²^+^ release inhibitors 2-APB and dantrolene. Mitochondrial Ca²^+^ was differentially reduced, with 2-APB and dantrolene causing the strongest inhibition (Fig. 5F through H), indicating that ER-derived Ca²^+^ is the main contributor.

Based on previous findings that the IBV 3a protein promotes ER Ca²^+^ efflux, we hypothesized that 3a mediates mitochondrial Ca²^+^ overload during IBV infection. To test this, CEK cells were infected with rSD-Δ3a or rSD-3a-GTG, recombinant viruses lacking functional 3a. Confocal imaging and flow cytometry revealed significantly reduced mitochondrial Ca²^+^ levels in cells infected with either mutant virus compared to the parental rSD strain (Fig. 5I through L). Our findings reveal that IBV 3a promotes ER Ca²^+^ efflux, contributing to mitochondrial calcium overload during infection.

### The IBV 3a protein promotes mitochondrial ROS accumulation and activates the NLRP3 inflammasome

**Fig 6:**
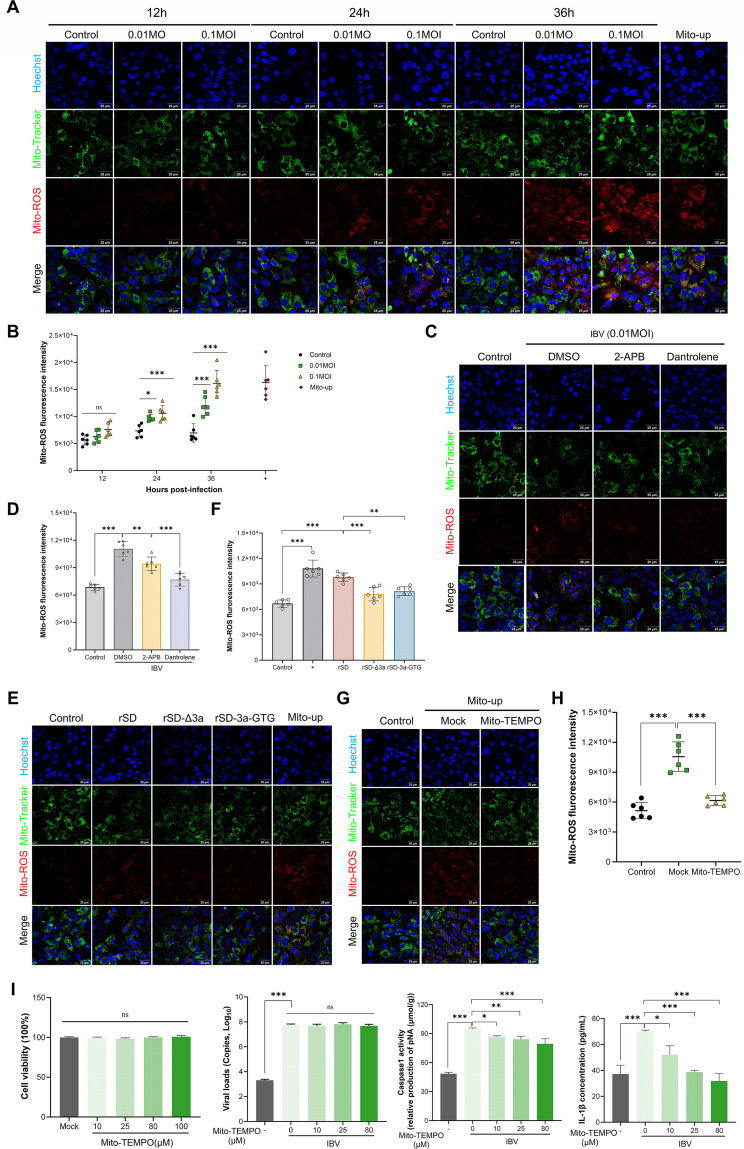
Effects of IBV infection on mitochondrial ROS levels and NLRP3 inflammasome activation. (**A and B**) CEK cells were infected with IBV and stained with a mitochondria-targeted ROS probe. Mitochondrial ROS levels were visualized by confocal laser scanning microscopy (**A**) and quantitatively analyzed at multiple time points and MOIs using a multi-mode microplate reader (**B**). (**C and D**) CEK cells infected with IBV were treated with 2-APB or dantrolene before staining with a mitochondrial ROS probe. Signal intensity was assessed by confocal microscopy (**C**) and quantified using a multi-mode microplate reader (**D**). (**E and F**) CEK cells infected with recombinant IBV strains were stained with the Mito-ROS probe. Mitochondrial ROS levels were visualized by confocal microscopy (**E**) and quantified using a microplate reader (**F**). (**G and H**) CEK cells were treated with the mitochondrial ROS inducer Mito-up, alone or with the scavenger Mito-TEMPO, to assess ROS scavenging efficacy. Mitochondrial ROS was visualized by fluorescence microscopy (**G**) and quantified by fluorescence intensity (**H**). (**I**) Caspase-1 activity and IL-1β release were measured in IBV-infected CEK cells treated with a concentration gradient of Mito-TEMPO. ns, not significant; *, *P* < 0.05; **, *P* < 0.01; ***, *P* < 0.001.

Mitochondrial calcium overload drives dysfunction and ROS accumulation, which activates the NLRP3 inflammasome (40, 41). We found that IBV infection increases mtROS in CEK cells, as shown by confocal microscopy and confirmed by microplate analysis, with levels rising in a time- and MOI-dependent manner (Fig. 6A and B). Single-cell RNA sequencing (scRNA-seq) revealed impaired mitochondrial bioenergetics in renal epithelial cells, with gene set enrichment analysis indicating significant downregulation of oxidative phosphorylation. Key subunits of mitochondrial complexes (*NDUFV1, SDHC, COX7A2L*) and TCA cycle enzymes, such as *MDH2,* were transcriptionally suppressed (Fig. S4A and B), likely reducing electron transport efficiency and promoting ROS accumulation.

As the endoplasmic reticulum is a major source of mitochondrial calcium influx during IBV infection, we examined whether inhibition of ER calcium release could reduce mtROS accumulation. Inhibition of ER calcium release by 2-APB and dantrolene significantly decreased mtROS levels in IBV-infected CEK cells, indicating that ER-mediated calcium overload promotes mitochondrial ROS production (Fig. 6C and D).

Given that IBV 3a induces mitochondrial calcium overload, we hypothesized that it also promotes mtROS generation and NLRP3 inflammasome activation. CEK cells infected with 3a-deficient viruses showed significantly lower mtROS fluorescence than those infected with parental rSD (Fig. 6E and F), confirming the critical role of 3a in mtROS production. Treatment with the mitochondrial ROS scavenger Mito-TEMPO dose-dependently suppressed Caspase-1 activation and IL-1β release (Fig. 6G through I). Overall, these results indicate that the IBV 3a protein promotes ER calcium release and mitochondrial calcium overload, leading to mtROS accumulation and NLRP3 inflammasome activation.

## DISCUSSION

**Fig 7:**
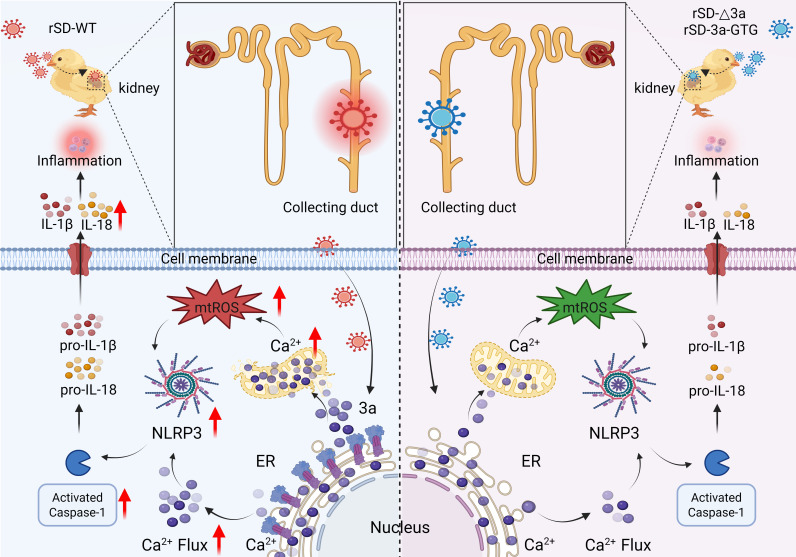
Schematic model of IBV 3a-driven NLRP3 inflammasome activation via the Ca²^+^–ROS axis, leading to collecting duct inflammation and kidney injury. Following IBV infection, the wild-type strain (rSD-WT) triggers Ca²^+^ release from the ER via the accessory protein 3a, promoting mitochondrial Ca²^+^ uptake and subsequent overload. This leads to the accumulation of mitochondrial reactive oxygen species (mtROS), and the combined elevation of Ca²^+^ and mtROS activates the NLRP3 inflammasome, resulting in Caspase-1 activation. Caspase-1 then cleaves pro-IL-1β and pro-IL-18 into their mature forms, promoting their release and driving renal inflammation and tissue injury. In contrast, 3a-deficient strains (rSD-Δ3a and rSD-3a-GTG) fail to effectively activate the Ca²^+^–ROS axis, leading to reduced cytokine production and attenuated renal inflammation. The schematic was created with BioRender (BioRender.com).

Excessive activation of the NLRP3 inflammasome is a critical driver of tissue injury during infection and cellular stress (21). Our previous work demonstrated that IBV-induced renal injury is mediated by NLRP3 inflammasome activation in collecting duct epithelial cells (33). However, the molecular mechanisms governing this process have yet to be fully elucidated. In this study, we further elucidate how IBV mediates renal inflammatory injury through NLRP3 inflammasome activation. We specifically identify the accessory protein 3a as a key viral effector that triggers ER calcium release, resulting in mitochondrial calcium overload and excessive mtROS production. These events ultimately activate the NLRP3–Caspase-1–IL-1β signaling axis, thereby promoting the release of pro-inflammatory cytokines and contributing to tissue injury progression (Fig. 7). These findings offer new molecular insights into the pathogenic mechanisms underlying IBV-induced renal inflammation.

Our previous study demonstrated that IBV infection triggers activation of the NLRP3 inflammasome in primary chicken embryonic kidney (CEK) cells, which in turn promotes the maturation and release of pro-inflammatory cytokines, particularly IL-1β and IL-18 (33). Notably, there are profound differences between avian and mammalian species in both the composition of inflammasome components and their activation mechanisms (42). In mammals, activation of NLRP3 typically involves oligomerization and aggregation into punctate structures near the nucleus, transitioning from an initially diffuse cytoplasmic distribution (43). These aggregates function as spatial platforms for the recruitment of downstream inflammasome components, such as ASC (apoptosis-associated speck-like protein containing a CARD) and Caspase-1, and are widely recognized as a hallmark of inflammasome activation (44). Among the hallmarks of inflammasome activation, the formation of ASC specks represents a key morphological marker of inflammasome assembly (45). However, previous studies have demonstrated that the chicken genome does not encode ASC, thereby preventing the production of this essential adaptor protein (42). This species-specific absence has two major implications: first, ASC speck formation is not applicable as a morphological marker of inflammasome activation in avian cells; second, the lack of ASC hinders the reconstitution of a fully functional chicken NLRP3 inflammasome *in vitro*. Despite the absence of ASC, avian NLRP3 has been shown to mediate IL-1β maturation and release (46–48). This indicates that NLRP3 may initiate inflammasome signaling in chickens via ASC-independent mechanisms, likely involving oligomerization and direct interaction with Caspase-1. Our previous experimental data suggest that, in the absence of ASC, punctate perinuclear NLRP3 aggregates in chicken cells may serve as an indicator of NLRP3 activation (33). These structures likely serve as a morphological marker for inflammasome activation under ASC-deficient conditions.

Using confocal fluorescence microscopy, we visualized the formation of perinuclear punctate NLRP3 aggregates in cells, serving as a morphological indicator of NLRP3 activation. This morphological feature was used as a screening readout to assess the regulatory potential of IBV-encoded proteins in inflammasome activation. Among the proteins tested, only the accessory protein 3a markedly induced this activation-associated phenotype upon co-expression with NLRP3, indicating its potential role in inflammasome regulation. Co-immunoprecipitation and pull-down assays revealed a direct interaction between 3a and NLRP3, whereas no association with Caspase-1 was detected, indicating that 3a acts as an upstream regulator in the NLRP3 inflammasome pathway. Structure–function analysis using truncation mutants indicated that 3a associates with several functional regions of NLRP3, including the NACHT, NLRC4-HD2V-like, and PPP1R42-like domains. These interactions are likely to perturb the autoinhibited conformation of NLRP3, thereby enhancing its oligomerization and promoting inflammasome assembly.

In addition to its role in the assembly phase of the NLRP3 inflammasome (signal 2), our western blot results suggest that 3a also influences the priming phase (signal 1). Specifically, 3a deletion reduced the expression of NLRP3 and pro-IL-1β, key components regulated by the NF-κB pathway, indicating that 3a may modulate NF-κB-driven upregulation of these proteins. This finding suggests that 3a contributes to both inflammasome priming and assembly, potentially enhancing the inflammatory response. Further studies on 3a’s effect on NF-κB signaling could provide insights into its dual role in NLRP3 activation and IBV-induced renal injury.

To examine the role of 3a in NLRP3 activation and IBV-induced renal pathology *in vivo*, we generated two 3a-deficient recombinant IBV strains (rSD-Δ3a and rSD-3a-GTG) using a reverse genetics system. Compared with the wild-type virus, both recombinant strains maintained similar replication efficiency but markedly suppressed NLRP3 inflammasome activation and proinflammatory cytokine expression, resulting in reduced renal injury and milder clinical symptoms in infected chickens. Collectively, these findings indicate that 3a contributes to IBV-induced pathology through dysregulation of host inflammatory responses, rather than by directly enhancing viral replication efficiency. This observation is consistent with the findings of Laconi et al., who demonstrated that 3a-deficient IBV strains exhibit reduced virulence (49), thereby reinforcing the hypothesis that 3a contributes to pathogenesis by hyperactivating the host immune response. Notably, the SARS-CoV-2 accessory protein ORF3a has been reported to activate the NLRP3 inflammasome, resulting in inflammatory cytokine production and immune-mediated tissue injury in multiple organ systems (28). These findings suggest that 3a-like proteins in coronaviruses may contribute to immunopathology via evolutionarily conserved inflammasome-activating mechanisms. Similar regulatory patterns have been documented in other viral infections, such as influenza, suggesting that targeting NLRP3 activation may represent a convergent strategy for viruses to modulate host immune responses and disease progression (19). These findings underscore the potential of targeting inflammasome pathways as a strategy for viral attenuation, as evidenced by specific envelope mutations in an attenuated equine infectious anemia virus vaccine strain that suppressed NLRP3 activation while preserving immunogenicity (50). This observation suggests a potential mechanism through which viruses modulate host inflammatory responses to maintain a delicate balance between immunoprotection and pathogenicity. In summary, IBV accessory protein 3a acts as a key modulator of NLRP3 inflammasome activation and host inflammatory responses, thereby shaping viral pathogenicity. These findings lay the groundwork for the rational design of attenuated live vaccines targeting inflammasome-mediated immunopathology.

Viroporins are pore-forming viral proteins that disrupt host ion balance and activate the NLRP3 inflammasome through ionic stress, as demonstrated in multiple viral infections (51). In SARS-CoV and SARS-CoV-2, the ORF3a protein facilitates potassium efflux, thereby delivering a canonical activation signal required for inflammasome assembly (28, 52). In parallel, the E protein depletes calcium reservoirs in the endoplasmic reticulum (ER) and Golgi apparatus, leading to mitochondrial stress and potentiation of NLRP3 activation (53). Similarly, the 2B proteins of encephalomyocarditis virus and human rhinovirus localize to ER and Golgi membranes, where they promote calcium efflux into the cytosol, ultimately triggering NLRP3 inflammasome activation (54, 55). Among avian viruses, both the influenza virus M2 protein and the 2B protein of duck hepatitis A virus type 1 (DHAV-1) have been reported to modulate intracellular calcium homeostasis (30, 56). DHAV-1 2B has been shown to both mobilize intracellular calcium and activate NF-κB signaling, thereby contributing to the dual-phase regulation of NLRP3 inflammasome priming and activation. The cooperative effect of potassium efflux and calcium mobilization constitutes a core ionic mechanism that sustains inflammasome activity and amplifies the downstream inflammatory response. In this study, we demonstrate that NLRP3 activation during IBV infection is contingent upon potassium efflux and ER calcium release, a conserved mechanism shared among various coronaviruses. Confocal imaging revealed that 3a-induced NLRP3 puncta colocalize with the ER, consistent with previous findings that IBV 3a predominantly localizes to ER-associated subcellular compartments (57). Further analysis revealed that 3a promotes ER calcium release, which triggers NLRP3 inflammasome activation and induces mitochondrial calcium overload. This overload leads to the accumulation of mtROS, further amplifying inflammasome activation. We propose that 3a may interact with specific domains of NLRP3 to facilitate its recruitment and anchoring to specific ER microdomains. Within this signal-rich microenvironment, 3a-induced calcium release and subsequent mtROS production act synergistically to drive NLRP3 activation. However, it remains unclear whether 3a functions as a bona fide viroporin or indirectly regulates calcium flux through interactions with endogenous calcium channels, such as inositol 1,4,5-trisphosphate receptors (IP3Rs) or ryanodine receptors (RyRs).

Taken together, our results demonstrate that the accessory protein 3a of IBV promotes NLRP3 inflammasome assembly through direct interaction with NLRP3. This interaction induces calcium release from the ER and promotes accumulation of mtROS, thereby enhancing activation of the NLRP3–Caspase-1–IL-1β axis and facilitating IL-1β release. Collectively, these events contribute to renal inflammatory injury, thereby elucidating a key molecular mechanism that underpins IBV-induced renal immunopathology in chickens.

## MATERIALS AND METHODS

### Viruses and cells

Three recombinant viruses were constructed using reverse genetics: rSD-Δ3a (with a deletion of the 3a gene), rSD-3a-GTG (harboring a point mutation in the 3a gene), and the parental recombinant virus rSD. The rSD-Δ3a virus was generated by deleting the entire 3a gene, effectively removing its expression. In contrast, the rSD-3a-GTG mutant was created by substituting the start codon of the 3a gene (ATG to GTG), which blocks translation initiation and prevents the expression of the 3a protein without altering the gene sequence itself. All recombinant viruses were generated in this study and are maintained in our laboratory. BHK-21 and HEK-293T cell lines were cultured in Dulbecco’s Modified Eagle Medium (Gibco, USA) supplemented with 10% fetal bovine serum (FBS; Gibco, USA) and 1% penicillin-streptomycin (Gibco, USA). HD11 cells were maintained in RPMI 1640 medium (Gibco, USA) supplemented with 10% FBS and 1% penicillin-streptomycin. Chicken embryo kidney (CEK) cells were isolated from 18-day-old SPF chicken embryos. All cells were incubated at 37°C in a humidified atmosphere containing 5% CO₂.

### Reagents and antibodies

Chicken IL-1β ELISA kit (SEA563Ga) was purchased from Cloud-Clone Product, China. Total RNA Isolation Kit was purchased from Magen, Beijing, China. M5 HiPer Real-Time PCR Super Mix was purchased from Mei5bio, Beijing, China. Lipofectamine 2000 Transfection Reagent (11668019) was purchased from Invitrogen. Caspase 1 Activity Assay Kit, Immunol Staining Fix Solution, Immunostaining Permeabilization Buffer containing Triton X-100, Immunol Staining Blocking Buffer, CCK-8 solution, Cell Lysis Buffer for Western and IP, Mito-Tracker Green, ER-Tracker Red, Mito-ROS, and Hoechst were purchased from Beyotime, China. Flag Nanoab Magarose Beads (FNM-25-500) and HA Nanoab Magarose Beads (HNM-25-500) were purchased from NuoyiBio, China. Glutathione Sepharose beads (GE Healthcare, Uppsala, Sweden). EGTA tetrasodium (Selleck, E4448), 2-aminoethyl diphenylborinate (2-APB) (Selleck, S6657), BAPTA-AM (Selleck, S7534), dantrolene sodium (Selleck, S5478), TG (Selleck, S7895). These inhibitors were used to investigate calcium signaling in NLRP3 inflammasome activation: EGTA tetrasodium prevents extracellular Ca²^+^ influx; 2-APB blocks IP3 receptors and Store-Operated Calcium Entry to inhibit ER Ca²^+^ release; BAPTA-AM chelates intracellular Ca²^+^ to differentiate influx from ER release; dantrolene sodium inhibits ryanodine receptors on the ER to block ER Ca²^+^ release; thapsigargin depletes ER Ca²^+^, promoting its release into the cytosol. Antibodies were Mouse mab IBV-N protein (HyTes, 3BN1), Rabbit pab NLRP3 (ABclonal, A24297), Rabbit pab IL1β (ABclonal, A16288), Rabbit pab Cleaved-Caspase1 (Wanlei Biotechnology, WL03450), Mouse mab β-actin (ABclonal, AC004), Mouse mab GAPDH (ABclonal, AC033), Rabbit pab GRP78 (ABclonal, A11366), Rabbit pab GOLGA4 (ABclonal, A10216). Alexa Fluor 488-conjugated anti-mouse IgG (H+L) (Cell Signaling Technology, 4408), Alexa Fluor 555-conjugated anti-rabbit IgG (H+L) (Cell Signaling Technology, 4413), Alexa Fluor 647-conjugated anti-mouse IgG (H+L) (Cell Signaling Technology, 4410).

### Plasmid construction and transfection

The full-length 3a gene was amplified from SD (GenBank: KY421673) cDNA and cloned into the pRK5-Flag vector. Other IBV proteins were generated following the same cloning procedure. The coding sequences of NLRP3 (GenBank accession no. AJ851656.1) and Caspase-1 (GenBank accession no. XM_040687588.2) were amplified from primary CEK cells and cloned into the pCAGGS expression vector fused with either an HA or Myc epitope tag. Truncated variants of NLRP3 were generated by PCR and similarly cloned into pCAGGS. All plasmid constructs were verified by Sanger sequencing. Expression plasmids were transfected into cultured cells using Lipofectamine 2000, following the manufacturer’s protocol. The coding sequence of G-CEPIA1er was synthesized based on a previously reported design (58) and inserted into the pCAGGS vector (Qingke, Beijing). For GST pull-down assays, the cytoplasmic (non-transmembrane) region of the 3a protein was cloned into the bacterial expression vector pGEX-6P-1. NLRP3 was subcloned into the pET-28a-His vector.

### Generation of mutant viruses

Virus rescue experiments were performed as previously described (59). Briefly, BHK-21 cells were seeded in six-well plates and cultured to confluence (approximately 10⁶ cells per well). Each well was transfected with 2.5 μg of the IBV YAC-BAC shuttle plasmid using Lipofectamine 2000 (Thermo Fisher Scientific). After 4–6 h, the transfection mixture was replaced with DMEM supplemented with 2% fetal bovine serum and 1% penicillin-streptomycin. At 48 h post-transfection, cells were frozen at –80°C and defined as passage 0 (P0). After three freeze-thaw cycles, the resulting lysates were inoculated into the allantoic cavities of 9- to 11-day-old SPF chicken embryos. Allantoic fluid was harvested for subsequent analysis. To assess the genetic stability of the rescued virus, Sanger sequencing was performed on the virus stock after seven passages in chicken embryos. The sequencing results confirmed the intended mutations in the GTG and Δ3a mutants, with no reversion to the wild-type sequence (Fig. S5).

### Enzyme-linked immunosorbent assay

Measurement of IL-1β concentration in cell supernatants and serum was performed using enzyme-linked immunosorbent assay (ELISA), according to the instructions provided by the manufacturer. A total of 100 μL of standards or samples was added to each well of a 96-well plate pre-coated with specific antibodies, followed by a 1-h incubation at 37°C. The plates were then emptied and gently dried with absorbent paper. Biotinylated anti-IL-1β antibody (100 μL) was added to each well and incubated for another hour at 37°C, after which the wells were washed three times. HRP-conjugated streptavidin (100 μL) was subsequently applied, followed by a 30-min incubation and five washes. Colorimetric detection was initiated with 90 μL of TMB substrate in the dark at 37°C for 10–20 min. The reaction was terminated by the addition of 50 μL stop solution, and optical density was measured at 450 nm.

### Caspase-1 activity assay

The enzymatic activity of Caspase-1 in cell lysates was measured using a commercially available kit (Beyotime, China). A total of 50 μL of lysate was mixed with 40 μL of reaction buffer, followed by the addition of 10 μL Ac-YVAD-pNA (2 mM) to make up 100 μL. Lysis buffer was used as the blank control. After incubation at 37°C until color development became apparent, absorbance was read at 405 nm.

### Real-time quantitative PCR

Total RNA was extracted from cells or tissues using an RNA extraction kit and reverse-transcribed to generate cDNA. The resulting cDNA was amplified by real-time quantitative PCR (RT-qPCR) using the M5 HiPer Real-Time PCR Super Mix. The expression levels of target genes were normalized to β-actin mRNA expression.

### Indirect immunofluorescence assay and confocal microscopy

Cell samples were harvested at designated times following transfection or infection and subsequently fixed using Immunol Staining Fix Solution. Permeabilization was carried out with Immunostaining Permeabilization Buffer containing Triton X-100, followed by blocking with Immunol Staining Blocking Buffer. The cells were then incubated with the appropriate primary antibodies at 4°C for 12 h. For fluorescent labeling, Alexa Fluor 488-conjugated anti-mouse IgG (H+L) and/or Alexa Fluor 555-conjugated anti-rabbit IgG (H+L) secondary antibodies were applied and incubated for 1 h at room temperature in the dark. Nuclear counterstaining was performed using DAPI for 10 min at room temperature. The cells were then washed five times with phosphate-buffered saline (PBS) containing Tween 20 (PBST), with each wash lasting 5 min. Imaging was conducted using a Nikon A1 fluorescence microscope (Nikon, Tokyo, Japan). Colocalization between signals was analyzed using Fiji (ImageJ) software. The observed Pearson’s correlation coefficient [R(obs)] was calculated to assess the degree of colocalization, with values ranging from −1 (complete negative correlation) to +1 (complete colocalization), and 0 indicating no spatial correlation.

### Co-immunoprecipitation assay

After transfection with the indicated plasmids, cell pellets were collected at specified time points. Cells were lysed in 1 mL of lysis buffer suitable for western blotting and immunoprecipitation. The lysates were centrifuged at 14,000 × *g* for 10 min, and the supernatants were pre-cleared by incubation with blank Magarose Beads for 2 h at 4°C. The resulting supernatants were incubated with Flag or HA Nanoab Magarose Beads at 4°C for 2–4 h. Immunoprecipitates were washed five times with ice-cold low-salt lysis buffer, resuspended in 2× SDS loading buffer, heated at 100°C for 10 min, and subsequently analyzed by western blotting.

### Drug treatment and virus infection

CEK cells were seeded into culture plates and grown to 80%–90% confluence. Prior to infection with IBV at an MOI of 0.01, cells were pretreated with small-molecule inhibitors at the indicated concentrations for 2 h. Following pretreatment, the drug-containing medium was removed, and cells were infected with IBV for 2 h at 37°C to allow viral adsorption. After infection, the viral inoculum was discarded, and cells were washed twice with PBS. Fresh maintenance medium containing the corresponding concentrations of the inhibitors was then added, and cells were incubated for an additional 24 h at 37°C in a 5% CO₂ incubator. After treatment, cells or supernatants were collected for downstream analyses, including IL-1β and Caspase-1 activity assays, immunofluorescence, and flow cytometry. The inhibitors used in this study included EGTA, BAPTA-AM, 2-APB, dantrolene, and Mito-TEMPO, all of which were administered following the same treatment protocol. Stock solutions were prepared by dissolving each compound in dimethyl sulfoxide or sterile water. Solvent control groups were prepared using equivalent volumes of the respective solvents to account for solvent-related effects.

### Quantification of intracellular potassium levels

Intracellular potassium levels were determined by ICP-MS, as previously described (34).

### Ca^2+^ measurements

#### Cytosolic Ca²^+^ measurement

Cytosolic Ca²^+^ levels were quantified using a Fluo-8 Calcium Flux Analysis Kit (Abcam, ab112129), following a previously described method with minor modifications (60). At the indicated time points post-IBV infection, CEK cells were incubated with 100 μL of Fluo-8 dye-loading solution in a 96-well plate at 37°C for 30 min, followed by an additional 30-min incubation at room temperature. Fluorescence signals were observed using a Nikon fluorescence microscope (Nikon, Tokyo, Japan) or quantified using a SpectraMax iD3 multi-mode microplate reader (Molecular Devices, USA) at excitation/emission wavelengths of 490/525 nm.

#### Endoplasmic reticulum calcium release assay

ER Ca²^+^ release was assessed as previously described (61). Thapsigargin (TG), an inhibitor of ER Ca²^+^-ATPase, blocks the reuptake of Ca²^+^ into the ER, resulting in the accumulation of Ca²^+^ in the cytosol. Therefore, the TG-induced increase in cytosolic Ca²^+^ is indicative of ER Ca²^+^ release. Briefly, CEK cells preloaded with Fluo-8 were washed twice with calcium-free HBSS and resuspended in calcium-free HBSS. TG (1 μM final concentration) was added immediately before fluorescence measurement, and cytosolic Ca²^+^ levels were determined as described above.

#### Mitochondrial Ca²^+^ measurement

Following IBV infection or drug treatment, CEK cells were incubated with 5 μM Rhod-2 AM (Abcam, ab142780) at 37°C for 30 min in the dark. After incubation, the cells were washed twice with PBS and resuspended in 500 μL of Hanks’ Balanced Salt Solution (Beyotime). Mitochondrial Ca²^+^ levels were then immediately analyzed by flow cytometry using a BD LSRFortessa flow cytometer (BD Biosciences, USA) with excitation/emission settings of 549/578 nm. Alternatively, cells were directly observed under a Leica STELLARIS STED confocal laser scanning microscope (Leica, Germany).

### Mitochondrial ROS detection

Mitochondrial superoxide levels were assessed using the Mitochondrial Superoxide Detection Kit (Beyotime, S0061). CEK cells were either infected with IBV or pretreated with specified reagents for defined durations. The culture medium was then removed, and the Mito-ROS probe was diluted 1:1,000 to a final concentration of 5 μM, according to the manufacturer’s instructions. Cells were incubated with the working solution at 37°C in the dark for 30 min. After incubation, the working solution was removed, and the cells were washed twice with PBS. Cells treated with Mito-up, a mitochondrial ROS inducer included in the kit, served as a positive control. Fluorescence was measured using a SpectraMax iD3 multi-mode microplate reader (Molecular Devices, USA) at excitation/emission wavelengths of 396/610 nm; alternatively, fluorescence signals were visualized using a Leica STELLARIS STED confocal laser scanning microscope (Leica, Germany).

### Live-cell imaging

BHK cells were seeded in glass-bottom dishes and transfected with plasmid DNA upon reaching 60%–70% confluency. Live-cell imaging was performed 24 h post-transfection. Following TG treatment, time-lapse live-cell imaging was conducted using a Leica STELLARIS STED confocal laser scanning microscope (Leica, Germany). To minimize photobleaching, the excitation laser intensity was set to 10%–20% of the maximum output. Control cells were imaged using the same parameters. Images were acquired using a 100× oil immersion objective and subsequently processed and analyzed using ImageJ software.

### *In vivo* infection experiments and sample collection

One-day-old SPF chickens in the three IBV-infected groups (rSD, rSD-Δ3a, and rSD-3a-GTG) were inoculated with 10^5.5^ TCID₅₀ of their respective viruses via the intranasal and ocular routes, while control chickens received an equal volume of PBS through the same routes. All chickens were monitored daily throughout the experimental period for clinical manifestations, including sneezing, tracheal rales, and drowsiness. At specific time points post-infection, three birds from each group were randomly chosen for euthanasia and necropsy. Macroscopic changes in the trachea, lungs, and kidneys were examined and recorded. Peripheral blood and tissues were aseptically collected. Tissues intended for immunohistochemical or histological examination were fixed in 10% neutral-buffered formalin. Lesion grading was performed following the criteria established in prior studies (62). The remaining tissue samples were rapidly frozen in liquid nitrogen and preserved at –80°C for subsequent analysis of gene expression and protein levels. Blood specimens were centrifuged to separate the serum, which was then used to quantify cytokine levels via ELISA.

### Single-cell transcriptomic analysis

We utilized a processed single-cell RNA sequencing (scRNA-seq) data set from our prior work, which profiled kidney tissues from IBV-infected and control chickens. The initial data processing, including quality control, normalization, dimensionality reduction, clustering, and cell type annotation, was performed as described (63).

For downstream analysis, we isolated collecting duct (CD) cells based on AQP2 expression. Differential gene expression between infected and control CD cells was analyzed using Seurat (v4.3.0). GSEA was performed to identify pathways influenced by IBV infection, using a pre-ranked gene list sorted by log₂ fold-change values. Enrichment analysis was conducted with the gseGO function from clusterProfiler (v4.6.0) against the GO database. Pathways with *P* < 0.05 were considered significant, and heatmaps of leading-edge gene expression were plotted using pheatmap.

### Statistical analysis

All data were analyzed using GraphPad Prism Software (version 9.0; San Diego, CA, USA). Comparisons between two groups were performed using Student’s *t*-test, and differences among multiple groups were assessed using one-way or two-way ANOVA. All experiments were independently repeated at least three times. Quantitative data are expressed as mean ± standard deviation (SD). *P* > 0.05 stands for not significant (ns); *, *P* < 0.05; **, *P* < 0.01; ***, *P* < 0.001 indicated in the figure captions.

## Data Availability

All relevant data are provided as figures within the paper and its supplemental material. Sequencing data have been deposited in Genome Sequence Archive (GSA) of National Genomics Data Center under the accession number CRA015620.

## SUPPLEMENTAL MATERIAL

Supplemental figures jvi.02125-25-s0001.docx Fig. S1 to S5.

Movie S1 jvi.02125-25-s0002.mp4 Real-time confocal imaging of BHK cells expressing G-CEPIA1er.

Movie S2 jvi.02125-25-s0003.mp4 Real-time confocal imaging of BHK cells expressing G-CEPIA1er following treatment with 1 μM thapsigargin (TG).

Movie S3 jvi.02125-25-s0004.mp4 Real-time confocal imaging of BHK cells transfected with an EGFP-expressing control plasmid.

Movie S4 jvi.02125-25-s0005.mp4 Real-time confocal imaging of BHK cells transfected with an EGFP-expressing control plasmid following treatment with 1 μM thapsigargin (TG).

ASM does not own the copyrights to Supplemental Material that may be linked to, or accessed through, an article. The authors have granted ASM a non-exclusive, world-wide license to publish the Supplemental Material files. Please contact the corresponding author directly for reuse.
