# Supplementary material for: Infectious bronchitis virus accessory protein 3a induces renal inflammatory injury by activating the NLRP3 inflammasome via ER calcium mobilization and mitochondrial ROS production
Source: J Virol. 2026 Apr 14;100(5):e02125-25. doi: 10.1128/jvi.02125-25 (PMC13185645; doi:10.1128/jvi.02125-25)
Supplement: Supplemental figures — Fig. S1 to S5. [file jvi.02125-25-s0001.docx]

**Supplementary Material**

**
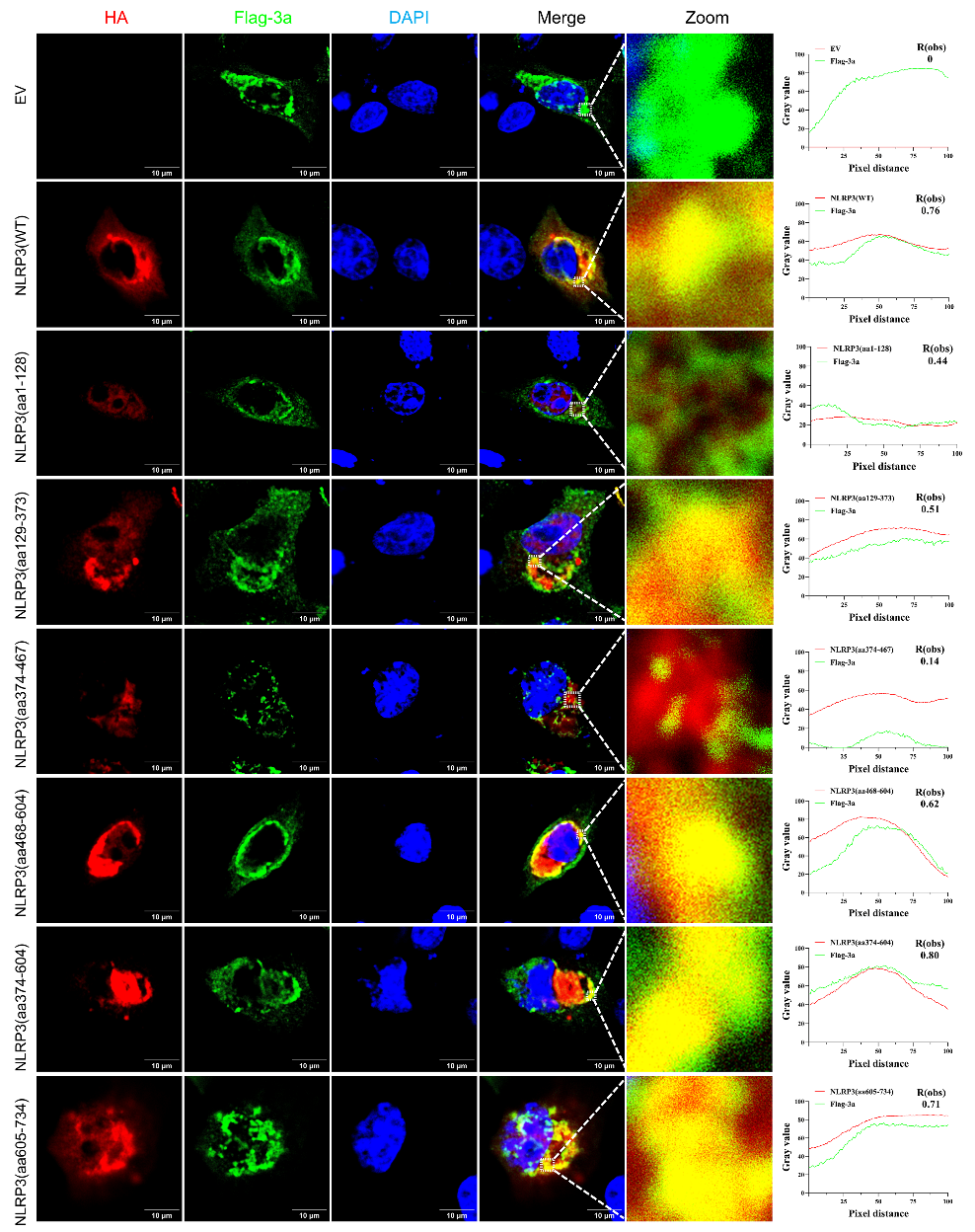
**

**FIG S1** Subcellular colocalization and aggregation analysis of full-length and truncated NLRP3 in response to IBV 3a expression. BHK cells were co-transfected with Flag-tagged IBV 3a protein and HA-tagged full-length NLRP3 (WT) or a series of NLRP3 truncation mutants, followed by incubation for 24 hours. Subcellular localization and aggregation were analyzed by confocal laser scanning microscopy. Cell nuclei were stained with DAPI (blue); NLRP3 truncation mutants were detected using the HA tag (red); and IBV 3a protein was visualized using the Flag tag (green). Scale bar = 10 μm.


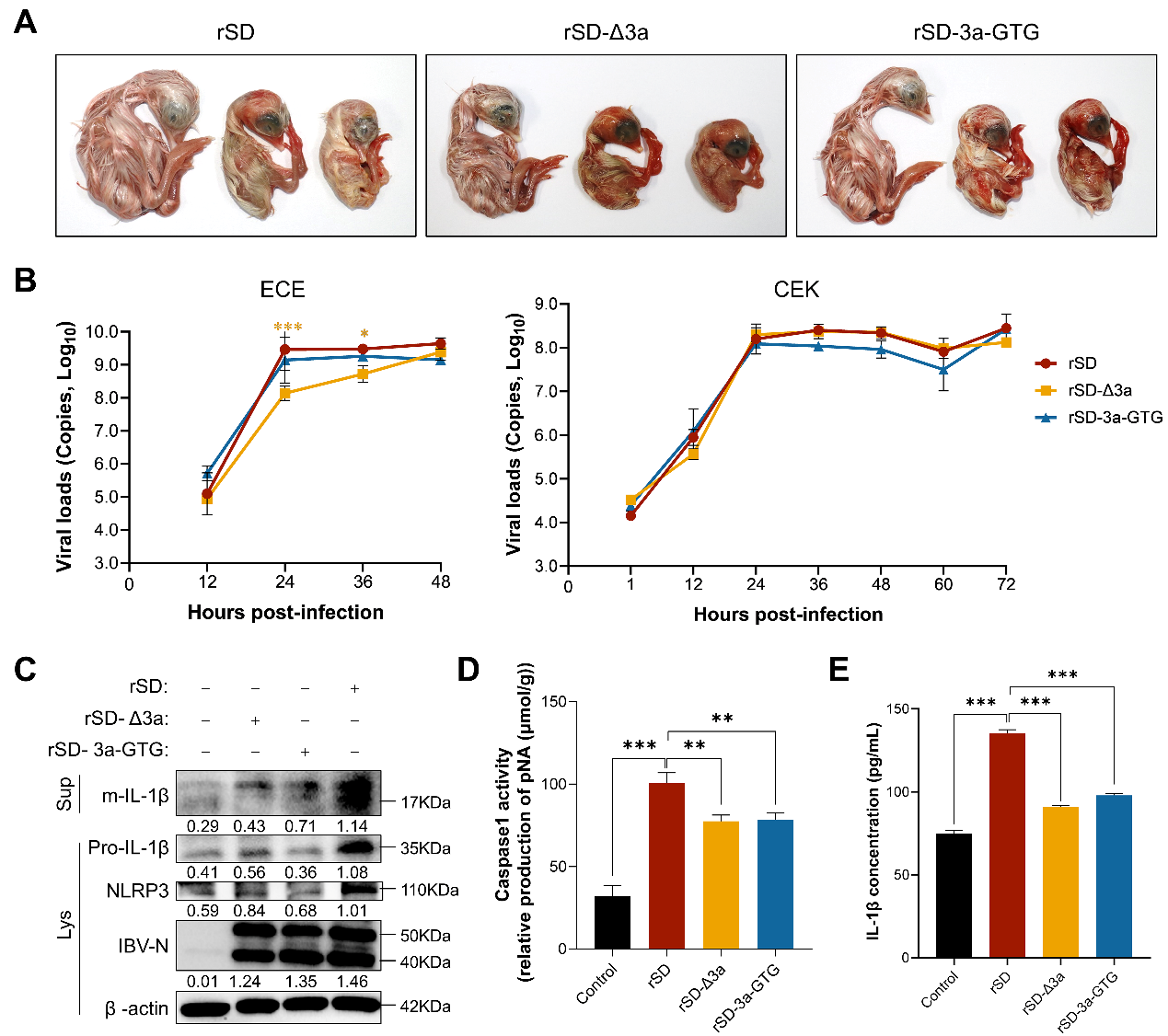


**FIG S2** Embryo lesions and replication kinetics of recombinant IBV strains. (A) Typical chicken embryo lesions caused by recombinant IBV strains rSD, rSD-Δ3a, and rSD-3a-GTG. (B) Comparative analysis of replication kinetics of recombinant viruses in ECEs and CEK cells. (C–E) Effects of loss of 3a protein expression on NLRP3 inflammasome activation in IBV-infected CEK cells. CEK cells were infected with recombinant viruses (MOI = 0.1) for 24 hours. Western blotting was performed to assess protein levels of NLRP3, pro-IL-1β, and mature IL-1β (C). Caspase-1 activity (D) and IL-1β release (E).


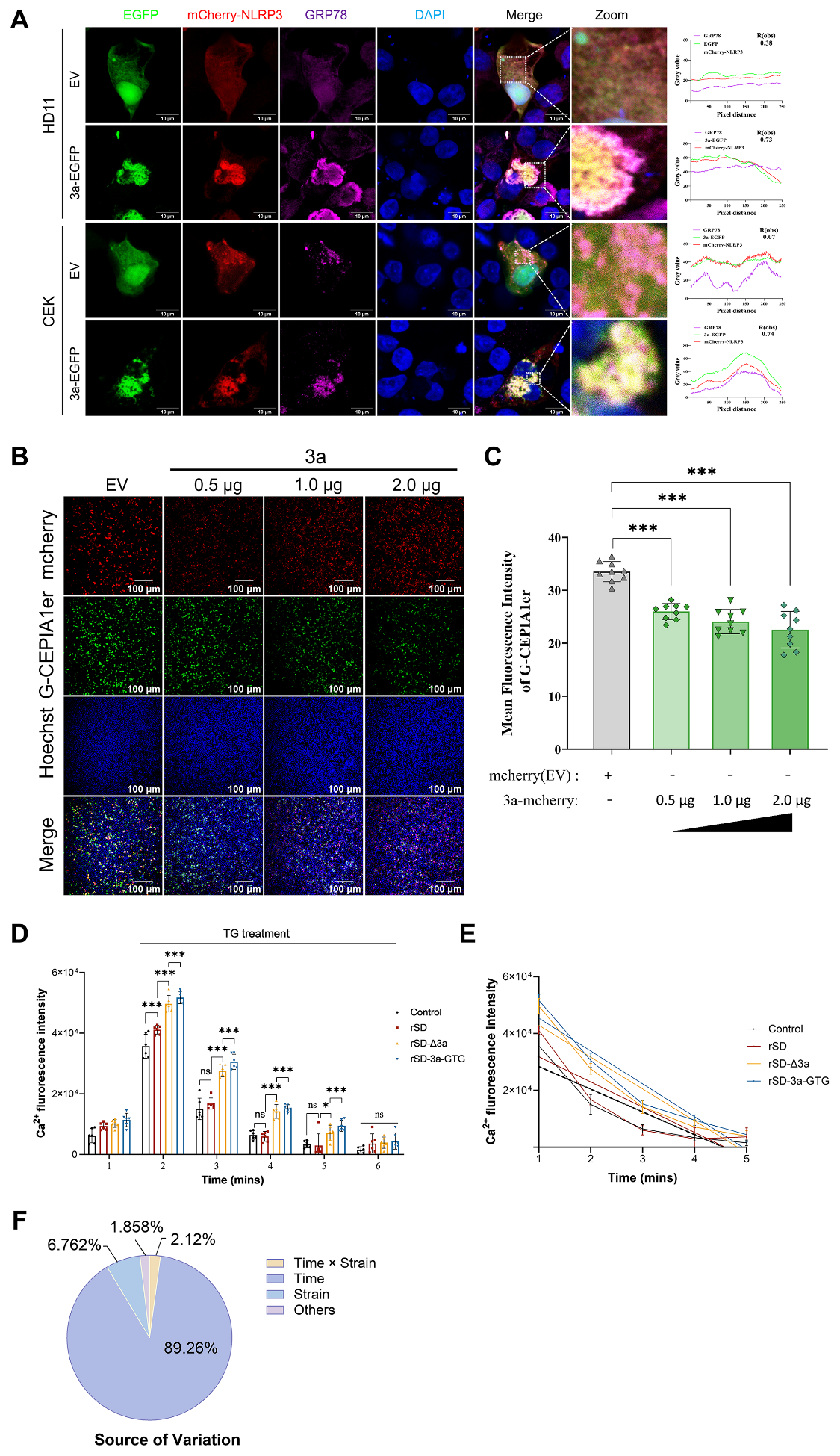


**FIG S3** IBV 3a protein disrupts endoplasmic reticulum calcium homeostasis and induces NLRP3 inflammasome activation. (A) Subcellular colocalization of 3a-EGFP and mCherry-NLRP3 in HD11 and CEK cells was assessed by confocal laser scanning microscopy. Cells were co-transfected with plasmids encoding 3a-EGFP and mCherry-NLRP3 and imaged 24 hours post-transfection to examine their subcellular distribution. GRP78 served as an ER marker. (B, C) G-CEPIA1er fluorescence was monitored and quantified in BHK cells co-expressing G-CEPIA1er and IBV 3a, transfected with increasing amounts of 3a plasmid. (D–F) CEK cells infected with different IBV strains (rSD, rSD-Δ3a, and rSD-3a-GTG) were stained with Fluo-8 AM. TG-induced ER calcium release was measured using a microplate reader at indicated time points (D). Two-way ANOVA was used to evaluate the effects of strain and treatment time on calcium dynamics (E, F).


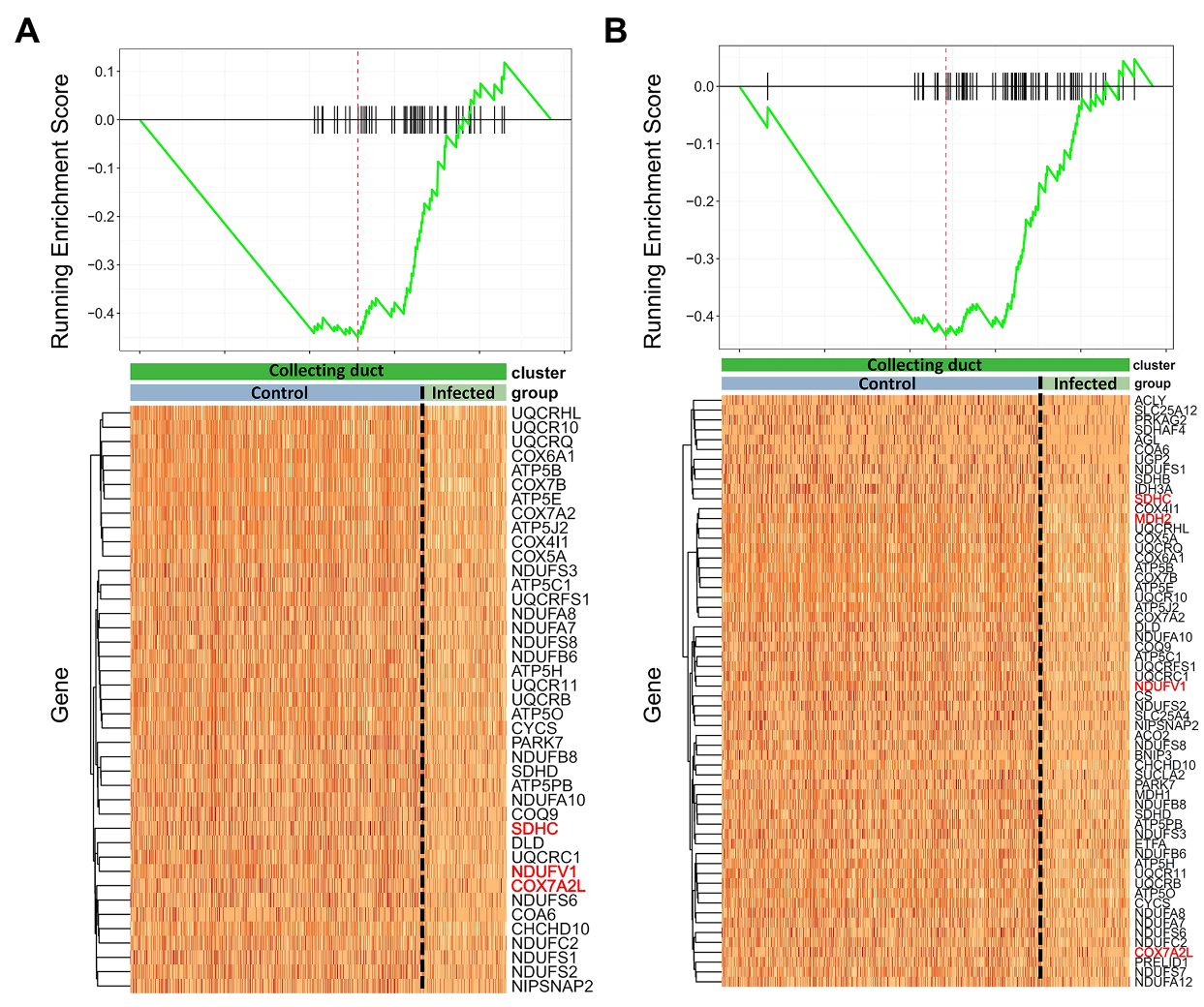


**FIG S4** Single-cell RNA sequencing (scRNA-seq) analysis of collecting duct cells from chicken kidneys on day 5 post-IBV infection. The upper panel displays results of gene set enrichment analysis (GSEA), identifying significantly enriched pathways in infected versus control groups, particularly those associated with oxidative phosphorylation (A) and energy metabolism derived from organic compound oxidation (B).


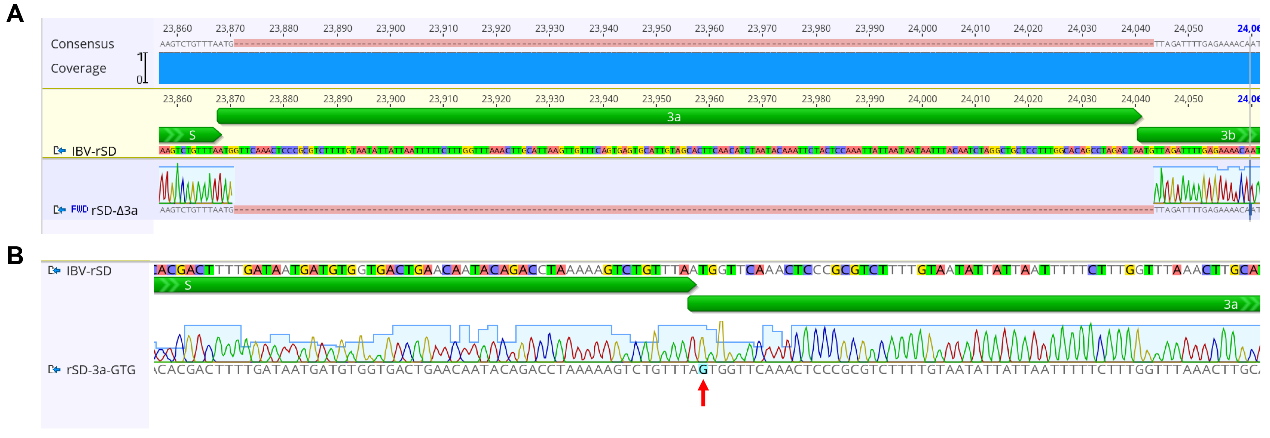


**FIG S5** Sanger sequencing analysis of the 3a gene regions in rescued virus stocks after seven passages in chicken embryos. (A) Sanger sequencing alignment of the 3a gene region in the rSD-Δ3a mutant, showing the expected deletion. (B) Sanger sequencing alignment of the 3a gene region in the rSD-3a-GTG mutant, confirming the intended start codon mutation (ATG to GTG). No reversion to the wild-type sequence was observed.**Movie 1.** Real-time confocal imaging of BHK cells expressing G-CEPIA1er. Images were acquired at 30 frames per second for 333.4 s. Playback is shown at 30 fps, ~83× real time.

**Movie 2.** Real-time confocal imaging of BHK cells expressing G-CEPIA1er following treatment with 1 μM thapsigargin (TG). Imaging was initiated immediately after TG addition to assess ER calcium dynamics. Images were acquired at 30 frames per second for 333.4 s. Playback is shown at 30 fps, ~83× real time.

**Movie 3.** Real-time confocal imaging of BHK cells transfected with an EGFP-expressing control plasmid. Images were acquired at 30 frames per second for 333.4 s. Playback is shown at 30 fps, ~83× real time.

**Movie 4.** Real-time confocal imaging of BHK cells transfected with an EGFP-expressing control plasmid following treatment with 1 μM thapsigargin (TG). Imaging was initiated immediately after TG addition. Images were acquired at 30 frames per second for 333.4 s. Playback is shown at 30 fps, ~83× real time.
